# Acetylmelodorinol isolated from *Sphaerocoryne affinis* seeds inhibits cell proliferation and activates apoptosis on HeLa cells

**DOI:** 10.1186/s12906-024-04357-w

**Published:** 2024-01-27

**Authors:** Nghia Le-Trung, Kenji Kanaori, Tomonori Waku, Thao Thi Phuong Dang, Kaeko Kamei

**Affiliations:** 1 Department of Functional Chemistry, Kyoto Institute of Technology, Kyoto, 606-8585 Japan; 2 Laboratory of Molecular Biotechnology, Faculty of Biology and Biotechnology, University of Science, Vietnam National University Ho Chi Minh City, Ho Chi Minh City, Vietnam

**Keywords:** Acetylmelodorinol, Anticancer, Anti-proliferation, Cervical cancer, HeLa cells, *Sphaerocoryne affinis*

## Abstract

**Background:**

Cervical cancer is a major global health concern with a high prevalence in low- and middle-income countries. Natural products, particularly plant-derived compounds, have shown immense potential for developing anticancer drugs. In this study, we aimed to investigate the anticancer properties of the pericarp and seeds of *Sphaerocoryne affinis* fruit on human cervical carcinoma cells (HeLa) and isolate the bioactive compound from the active fraction.

**Methods:**

We prepared solvent fractions from the ethanol extracts of the pericarp and the seed portion by partitioning and assessing their cytotoxicity on HeLa cells. Subsequently, we collected acetylmelodorinol (AM), an anticancer compound, from the ethyl acetate fraction of seeds and determined its structure using nuclear magnetic resonance. We employed cytotoxicity assay, western blotting, Annexin V apoptosis assay, measurement of intracellular reactive oxygen species (ROS) levels, 4′,6-diamidino-2-phenylindole (DAPI) staining, and a terminal deoxynucleotidyl transferase dUTP nick end labeling (TUNEL) assay, to evaluate the anticancer properties of AM on HeLa.

**Results:**

The solvent fractions from the seed displayed considerably higher cytotoxic activity against HeLa cells than those of the pericarp. We isolated and identified acetylmelodorinol as an anticancer compound from the ethyl acetate fraction from *S. affinis* seed extract. Treatment with acetylmelodorinol inhibited HeLa cell proliferation with an IC_50_ value of 2.62 ± 0.57 µg/mL. Furthermore, this study demonstrated that acetylmelodorinol treatment disrupted cell cycle progression by reducing the expression of cyclin E, CDK1/2, and AKT/mTOR pathways, increasing the intracellular ROS levels, reducing BCL-2/BCL-XL expression, causing DNA fragmentation and nuclear shrinkage, and triggering apoptosis through caspase 3 and 9 activation in a dose-and time-dependent manner.

**Conclusion:**

In contrast to previous reports, this study focuses on the inhibitory effects of AM on the AKT/mTOR pathway, leading to a reduction in cell proliferation in cervical cancer cells. Our findings highlight the promising potential of acetylmelodorinol as an effective treatment for cervical cancer. Additionally, this study establishes a foundation for investigating the molecular mechanisms underlying AM’s properties, fostering further exploration into plant-based cancer therapies.

**Supplementary Information:**

The online version contains supplementary material available at 10.1186/s12906-024-04357-w.

## Background

Cervical cancer remains a significant global health issue, primarily affecting women worldwide [1]. In 2020, an estimated 604,127 cervical cancer cases and 341,831 resultant deaths occurred globally, highlighting the ongoing burden of the disease [2], particularly in low- and middle-income countries (LMICs) [3]. Despite advancements in preventative strategies such as regular screening for high-risk human papillomavirus (HPV) infections and vaccination programs for HPV, the incidence and mortality rates of cervical cancer continue to be high, particularly in LMICs with limited resources for early detection and treatment [3]. Therefore, the development of novel, effective, and affordable anticancer therapeutics for cervical cancer remains critical in ongoing oncological research.

The development of therapies targeting apoptosis has long been a key focus in clinical oncology [4]. Apoptosis is regulated through two primary signaling pathways: the intrinsic pathway, activated by disturbances in the cell’s microenvironment, such as DNA damage and ROS, and kinase signaling pathways, such as AKT/mTOR [5, 6]. These events lead to enhanced permeabilization of the mitochondrial outer membrane (OMM) and the activation of caspases, specifically caspase 9 and caspase 3 [6]. The pro-apoptotic members of the BCL-2 family, including BAX and BAK, are responsible for OMM permeabilization, whereas the anti-apoptotic members, like BCL-2 and BCL-XL, inhibit this process [7, 8]. However, the extrinsic pathway, stimulated by death receptors, is mediated by caspase 8, eventually leading to the activation of executioner caspases, primarily caspase 3 [6].

Natural products, particularly those derived from plants, are valuable sources of bioactive compounds and have played a substantial role in drug discovery over the past several decades [9]. In anticancer drug research, plant-derived compounds have repeatedly proven to be a rich reservoir for potential therapeutic agents, with several plant-derived compounds, including taxol and vinblastine, successfully developed into clinically approved drugs [9]. One plant with potential anticancer properties is *Sphaerocoryne affinis*, a species belonging to the Annonaceae family that has been traditionally used in Southeast Asia as a remedy for various ailments [10, 11]. Previous studies investigating *S. affinis* have reported its antioxidant [12], anticancer, and antifungal properties [13], with some chemical constituents demonstrating considerable anticancer activities [13]. An extensive range of components from the SA tree, including heptenes, butenolides, triterpenoids, and flavonoids, have been widely reported as sources of anticancer substances [10, 14–18].

Our group also reported the anticancer activity of *S. affinis* fruits against human cervical carcinoma (HeLa) cells [19]. Among the reported constituents, acetylmelodorinol (AM), a heptene found in the stem bark [15, 17] and roots [10] of *S. affinis*, has garnered interest because of its bioactivity. Acetylmelodorinol, an example of γ-alkylidenebutenolides, has a stereoisomerically pure hydroxy group in its alkylidene segment, generating interest within the organic chemistry field owing to its simple and consistent structural characteristics [20]. Although previous studies have reported the cytotoxicity of AM against various cancer cell lines, such as human breast adenocarcinoma (MCF7), human hepatocellular carcinoma (HepG2), human cervical carcinoma (HeLa), and human epithelial carcinoma (KB), a comprehensive evaluation of its anticancer mechanisms and potential applications remains unexplored. A recent study by Thepmalee et al. [21] found that the AM isolated from the stem bark of *Xylopia pierrei* can enhance the expression of death receptors and their ligands, as well as activate apoptosis pathways in triple-negative breast cancer cells.

*Sphaerocoryne affinis* is a prospective candidate for anticancer investigations. This study primarily evaluates the anticancer properties of *S. affinis* fruit, specifically the pericarp (SAP) and seed (SAS) components, as potential sources of bioactive compounds. Furthermore, the objective is to identify and isolate the active components with anticancer properties from the most promising extract to examine their mode of action and potential cytotoxic effects on cervical cancer HeLa cells. This study aimed to broaden the understanding of the mechanistic properties of these anticancer compounds.

## Methods

### Materials and general methods

Fresh *S. affinis* fruit was purchased from a local market in Tay Ninh province, Vietnam, and identified by Dr. Dang Le Anh Tuan from the Laboratory of Botany, Department of Ecology and Evolutionary Biology, Faculty of Biology, Biotechnology, University of Science, Vietnam National University, Ho Chi Minh City, Vietnam. The voucher specimen PHH0004912 confirmed the identity of the plant, and the collection followed the IUCN Policy Statement on Research Involving Species at Risk of Extinction and Convention on Trade in Endangered Species of Wild Fauna and Flora.

We purchased ethanol, hexane, EA, and methanol from Fujifilm Wako Chemicals (Osaka, Japan). We obtained silica gel 60 with a size range of 0.040–0.063 mm for chromatography (230–400 mesh ASTM), and thin-layer chromatography silica gel 60 (aluminum supports) coated with fluorescent indicator F-254 from Merck (Darmstadt, Germany).

We cultured HeLa and OUMS-36 cells, obtained from the JCRB Cell Bank (National Institute of Biomedical Innovation, Health, and Nutrition, Japan) in specific media. While HeLa cells were cultured in Eagle's Minimum Essential Medium (Nacalai Tesque, Japan), OUMS-36 cells were cultured in Dulbecco's Modified Eagle Medium (Fujifilm, Tokyo, Japan). Both media were supplemented with 10% fetal bovine serum and 1% penicillin/streptomycin. The cells were incubated at 37 °C with 5% CO_2_ in a humidified environment.

### Preparation of *S. affinis* fruit extracts

Thirty kilograms of fresh *S. affinis* fruit were separated into pericarps and seeds and lyophilized. The dried pericarp (SAP, 770 g) and seeds (SAS, 608 g) were ground and soaked in ethanol for 7 d, and the supernatants were collected and evaporated. Extracts of SAP (209.2 g) and SAS (84.85 g) were obtained. The crude ethanolic extract was partitioned into EA, hexane (Hex), and water (Wat) fractions. Each fraction was concentrated using a rotary vacuum evaporator and lyophilized. Using 71.1 g of SAS ethanol extract, 45.4 g of SAS-Hex, 18.2 g of SAS-EA, and 6.3 g of SAS-Wat were obtained. Similarly, from 209 g of the SAP ethanol extract, 7.1 g of SAP-Hex, 7.9 g of SAP-EA, and 182.9 g of SAP-Wat were obtained.

### Isolation of anticancer compound from SAS-EA

The SAS-EA (12.05 g) was loaded onto a silica gel 60 column (2 × 70 cm), equilibrated with hexane, and eluted with a mixture of Hex–EA and EA–methanol. Nine fractions were obtained: E1–E7 (eluent: Hex:EA at 0:0, 9:1, 7:3, 7:3, 7:3, 4:6, and 0:10) and E8–E9 (eluent: EA:methanol at 1:9 and 0:10). The purity of each fraction was assessed using thin-layer chromatography on silica gel 60 F-254 plates. After fraction E4, eluted with a Hex:EA mixture of 7:3, was evaporated using a rotary evaporator, and the residue was dissolved in a small amount of EA. The mixture was then placed in a fume hood. Finally, an anticancer compound was obtained as a crystal during solvent evaporation. The yield of the compound was 3.9 g. This corresponds to the yield of 0.97% from total SAS powder.

### NMR analysis

Electrospray ionization time-of-flight (ESI-TOF) mass spectrometry (MS) spectra were measured using a micrOTOF spectrometer (Bruker, Faellanden, Switzerland), and the chemical structures of the isolated anticancer compounds were identified using one-dimensional (1D) and two-dimensional (2D) NMR spectra recorded on an AV-600 spectrometer (Bruker, Faellanden, Switzerland).

### Dose-dependent cytotoxicity assay

Cytotoxicity was assessed using the Cell Counting Kit-8 (CCK8) in HeLa cells (approximately 1 × 10^4^ cells/well) in 96-well plates. The cells were treated for 48 h with the solvent fractions or the anticancer compound dissolved in dimethyl sulfoxide (DMSO) at appropriate concentrations, maintaining DMSO in the culture medium at 0.25% (v/v). After treatment, the culture medium was replaced with a fresh medium containing CCK-8 solution (Dojindo Laboratories Kumamoto, Japan) in a 10:1 (v/v) ratio and incubated for 3 h at 37 °C. Absorbance was measured at 450 nm using a microplate reader (SH-1200, Corona Electric, Japan). Cytotoxicity was expressed as the percent inhibition of cell viability in the treatment groups, either with the extracts or the compound, compared with that of the non-treatment control group. Additionally, the IC_50_ or IC_90_ value was determined, which represents the concentration of the extract or the compound required to reduce the population of viable cells by 50% or 90%, respectively, compared with that of the non-treated group.

### Time-dependent cytotoxicity assay

Following the same protocol as that for the dose-dependent cytotoxicity assay, HeLa cells were treated with the extracts or the anticancer compound at IC_50_ or IC_90_ concentrations, and cell viability was assessed at various treatment times using the CCK8 assay.

### Clonogenic formation assay

HeLa cells (approximately 250 cells/well) were seeded in a 24-well plate and incubated with the compound dissolved in DMSO at IC_50_ or IC_90_ the following day. DMSO in the culture medium was maintained at 0.25% (v/v). After 36 h of treatment, the compound was removed from the medium, and the cells were cultured in a fresh compound-free medium. This process of culture medium replacement was repeated every two days for an additional 10 d. Finally, the cells were stained with 2% crystal violet, and colony formation was observed under a stereoscopic microscope (Olympus, Tokyo, Japan). A cluster of more than 50 cells was considered a colony.

### Terminal deoxynucleotidyl transferase–mediated dutp nick end labeling (TUNEL) assay

HeLa cells (approximately 1 × 10^4^ cells/well) in a 24-well plate were treated with AM at the IC_50_ concentration for 24 h and subjected to the TUNEL assay using the Apoptosis in Situ Detection Kit (Fujifilm, Tokyo, Japan), according to the manufacturer's instructions. The Peroxidase Stain DAB Brown Stain Kit (Nacalai, Kyoto, Japan) was used to detect apoptotic cells imaged under an IX70 microscope (Olympus, Tokyo, Japan).

### 4′,6-Diamidino-2-Phenylindole (DAPI) staining

HeLa cells (approximately 1 × 10^4^ cells/well) were seeded in a 24-well plate and incubated for 1 d. The cells were treated with the compound at the IC_50_ concentration for 24 h and then incubated with DAPI for 15 min. Fluorescence images were obtained using a FLUOVIEW FV10i confocal system (Olympus, Tokyo, Japan).

### Western blotting

HeLa cells (approximately 2.7 × 10^5^ cells/dish) were seeded in 35 mm dishes and treated with the compound for 12 h. Whole cell lysates were prepared using radioimmunoprecipitation assay (RIPA) buffer (Cell Signaling Technology, USA), and proteins were quantified using the Pierce BCA Protein Assay Kit (Thermo Fisher Scientific, US). Proteins (20 µg) were separated by 15% sodium dodecyl sulfate–polyacrylamide gel electrophoresis (SDS-PAGE) and transferred to a polyvinylidene difluoride membrane. After blocking with 5% bovine serum albumin, the membrane was incubated with primary antibodies at appropriate dilutions (Table S1), followed by incubation with secondary antibodies. The bands were visualized using Amersham ECL Prime Western Blotting Detection Reagent (GE Healthcare, US). The images were acquired using the Ez-Capture MG system (ATTO, Tokyo, Japan), and the intensity of bands was analyzed using a Gel Analyzer 19.1 (www.gelanalyzer.com).

### Annexin A5 apoptosis detection assay

Apoptotic cells were analyzed using the Annexin A5 Apoptosis Detection Kit (#640,914, Biolegend, USA) following the manufacturer's instructions. The procedure involved overnight culture of HeLa cells in 35 mm dishes at a density of 1 × 10^5^ cells/mL, followed by culture with medium containing IC_50_ (2.6 µg/mL) or IC_90_ (6.25 µg/mL) concentrations of AM. After 12 h of treatment with the compound, the cells were harvested using trypsin. Harvested cells were then washed once with Staining Buffer (Biolegend, USA) and resuspended in the Binding Buffer provided in the kit. The cells were stained with Annexin V and propidium iodide solution for 15 min at 25 °C and subsequently analyzed using the RF-500 flow cytometer (Sysmex, Hyogo, Japan).

### Measurement of intracellular ROS levels

Intracellular ROS levels were measured using a photo-oxidation-resistant DCFH-DA assay according to the manufacturer's instructions (#R253, Dojindo, Japan). HeLa cells were harvested using the same method mentioned in “Annexin A5 Apoptosis Detection assay.” The harvested cells were then washed with fresh medium and incubated with DCFH-DA at a final concentration of 10 nM in the loading buffer provided in the kit. After incubation for 30 min at 37 °C, the cells were washed thrice with the medium. The ROS levels were analyzed using an RF-500 flow cytometer, and the data were analyzed using FCSalyzer 0.9.22 (https://sourceforge.net/projects/fcsalyzer/).

### Data analysis

Experiments were performed in triplicate, and statistical analysis was performed using one-way analysis of variance (ANOVA) using GraphPad Prism 9 software. Dunnett’s post hoc test was used for pairwise comparisons with the control group. Data values are reported as mean ± standard deviation (SD), and significance was established at a *P*-value < 0.05 for both ANOVA and Dunnett’s test.

## Results

### Cytotoxic effects of SAP and SAS on Hela cells

**Fig. 1:**
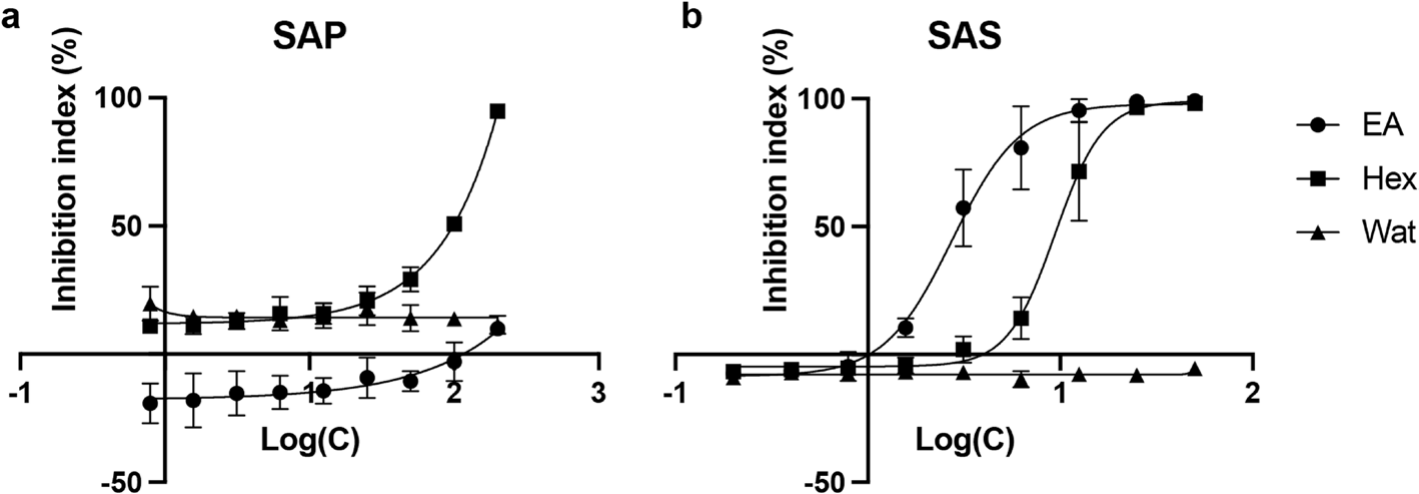
Cytotoxicity of solvent fractions from SAP and SAS extracts. HeLa cell viability after 48-h treatment with solvent fractions of SAP (**a**) and SAS (**b**) extracts were measured and expressed as percent inhibition of cell viability of treatment groups compared with that of the non-treatment control group. Significant cytotoxic effects were observed exclusively in the solvent fractions of SAS. Solvent fractions: circle, EA; square, Hex; triangle, water (Wat). X axis, the logarithm of the concentration (µg/mL) to the base 10. Data are represented as mean ± SD; *n* = 3

To explore the anticancer activities of SAP or SAS extracts, we assessed the cytotoxicity of the solvent fractions prepared by partitioning the ethanol extracts of SAP and SAS based on their effects on HeLa cells (Fig. 1). The results showed that none of the solvent fractions from the SAP extract exerted significant effects on cell viability. However, the Hex and EA fractions derived from the SAS extract displayed distinct cytotoxic effects, with calculated IC_50_ values of 8.15 and 2.76 µg/mL, respectively. Considering the higher cytotoxicity in the SAS-EA fraction than the SAS-Hex fraction, we selected the SAS-EA fraction for further investigation and isolation of potential anticancer compounds.

### Isolation and identification of an anticancer compound

**Fig. 2:**
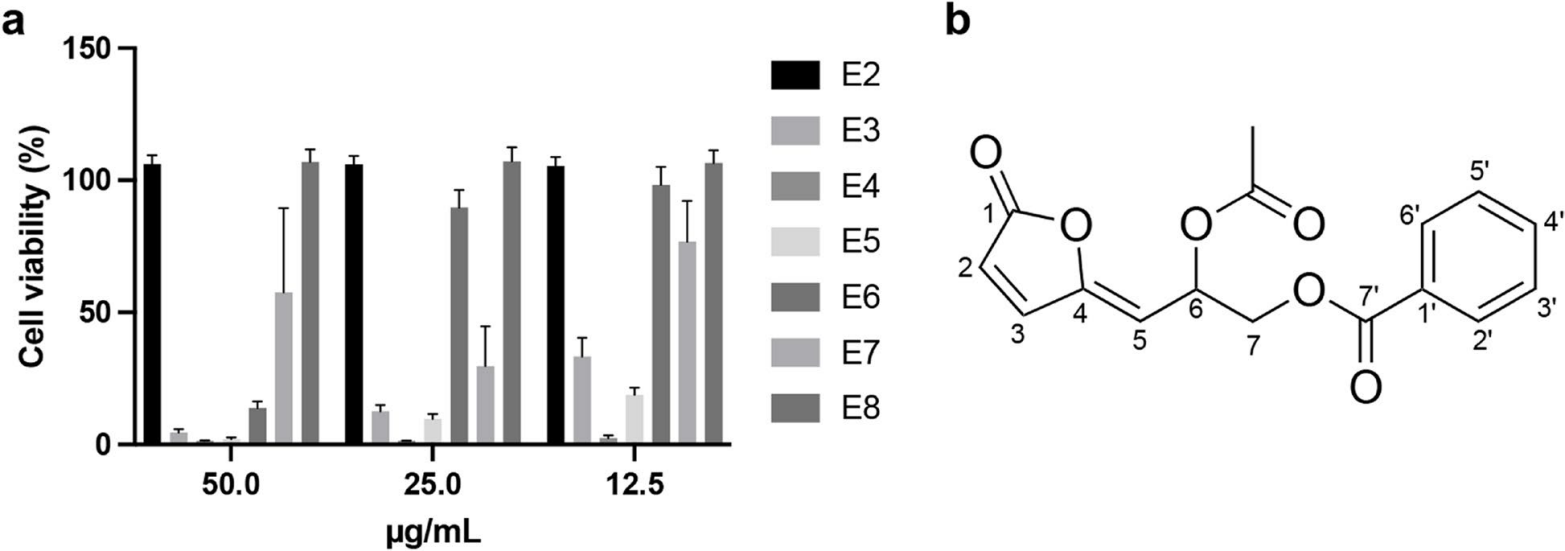
Isolation and identification of AM from SAS-EA. **a** Viability of Hela cells cultured with fractions E2 to E8 at concentrations of 50, 25, and 12.5 µg/mL for 48 h. E4 exhibited the strongest inhibitory effect on HeLa cell proliferation. AM was isolated from E4. **b** Chemical structure of AM was identified using ESI-TOF–MS. Data are represented as mean ± SD; *n* = 3

To identify potential compounds responsible for the observed cytotoxic effects of the SAS-EA fraction, we subjected the extract to silica gel chromatography, which separated nine individual fractions labeled E1–E9. Due to their limited yields, fractions E1 and E9 were not analyzed further. The data revealed that fraction E4 exhibited a significant inhibitory effect on the proliferation of HeLa cells (Fig. 2a). An anticancer compound was crystallized and purified from E4. The compound was analyzed using ESI-TOF–MS, which showed a positive peak at 325 [M + Na] + , corresponding to the chemical formula C16H14O6. The calculated molecular weight of the compound was determined to be 302. The ^1^H and ^13^C 1D-NMR, as well as 2D-NMR experiments including DQF-COSY, NOESY, ^1^H-^13^C HSQC, and HMBC spectra (Fig. S1–S6) confirmed that the compound was AM (Fig. 2b) [22].

### Cytotoxic effect of AM on Hela cells

**Fig. 3:**
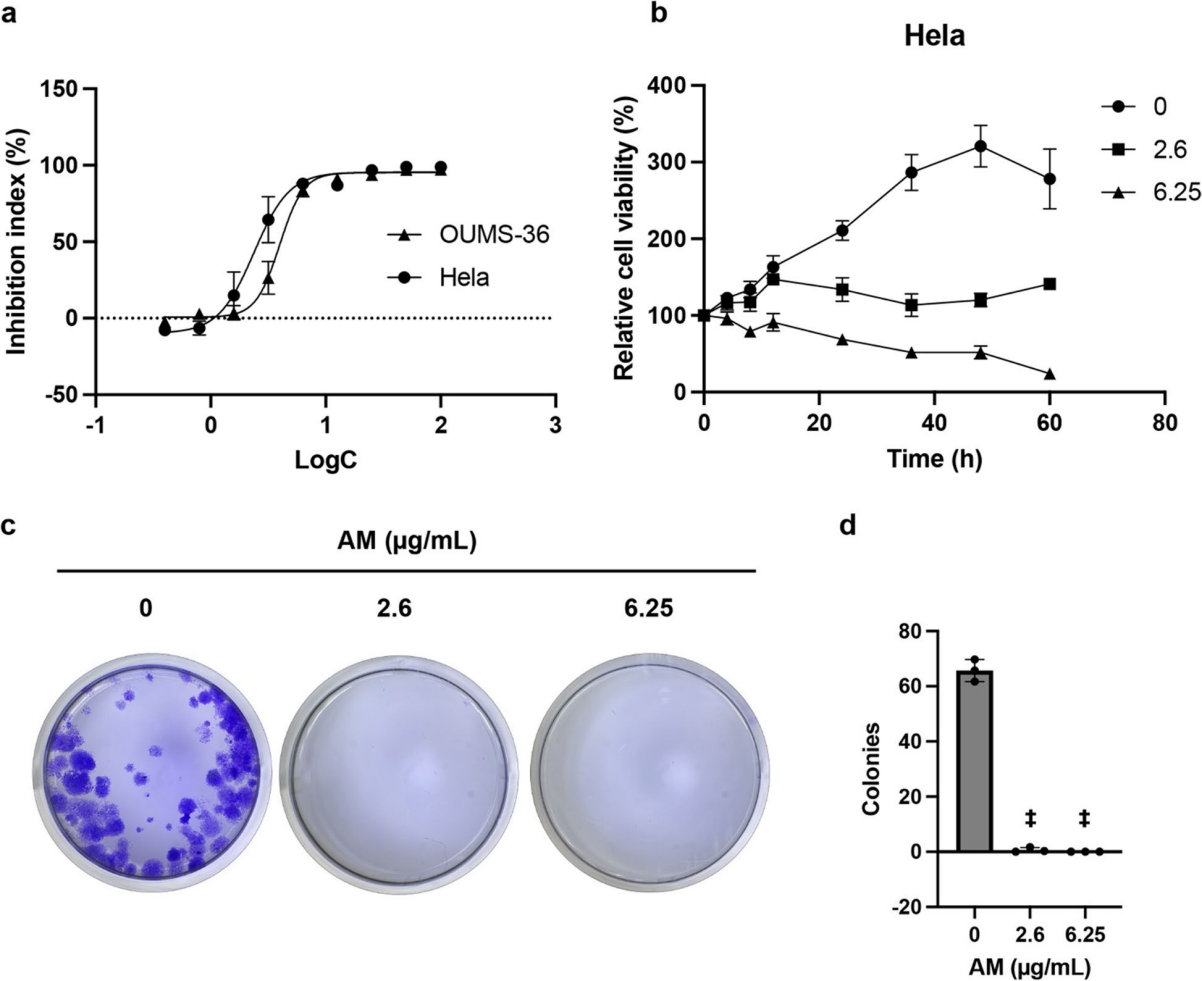
Cytotoxicity assessment of AM in HeLa cells. **a** The dose-dependent cytotoxicity of AM was assayed at concentrations ranging from 0.39 to 100 µg/mL against HeLa and OUMS-36 cells and expressed as percent inhibition of cell viability of treatment groups with AM compared with that of the non-treatment control group. X axis, the logarithm of the AM concentration (µg/mL) to the base 10. *n* = 3. Error bar, mean ± SD. **b** The time-dependent cytotoxicity on HeLa cells of AM at IC_50_ (square, 2.6 µg/mL) and IC_90_ (triangle, 6.25 µg/mL) concentrations for various times (0, 4, 8, 12, 24, 36, 48, and 60 h). Non-treatment group was used as a control (circle). Data are expressed as relative cell viability at each time to that at time 0. **c** Representative images of the clonogenic assay. HeLa cells were treated with AM at IC_50_ (2.6 µg/mL) and IC_90_ (6.25 µg/mL) concentrations for 36 h and then cultured in a compound-free medium for 10 d. Subsequently, the cells were stained with crystal violet and observed under a microscope. **d** Numbers of colonies containing over 50 cells formed by the 10-d culture in the clonogenic forming assay in panel (c). Data are represented as mean ± SD and statistically analyzed by one-way analysis of variance (ANOVA); Statistically significant differences were observed between the treated groups and the non-treated group; ‡, *p* < 0.0001; *n* = 3

To further investigate the cytotoxic effects of AM on HeLa cells, dose- and time-dependent assays were conducted. In the dose-dependent assay, the inhibitory effects of AM at concentrations ranging from 0.39 to 100 µg/mL on cell viability were evaluated on both HeLa and OUMS-36 cells, which were used as normal cells for comparison. The results indicated that the IC_50_ and IC_90_ values of AM against HeLa cells were 2.62 ± 0.57 µg/mL and 6.23 ± 1.49 µg/mL (Fig. 3a). However, AM displayed IC_50_ and IC_90_ values of 4.07 ± 0.48 and 7.61 ± 0.96 µg/mL, respectively, on OUMS-36 cells. In the time-based assay, the effects of AM on HeLa cell viability were examined over a range of durations. Cells treated with AM at both the IC_50_ and IC_90_ concentrations exhibited a decrease in viability after 12 h of treatment, with the effects of the IC_90_ concentration being evident from as early as 4 h onwards (Fig. 3b). A clonogenic forming assay was used to assess the colony-forming ability of HeLa cells after treatment with AM at the IC_50_ and IC_90_ concentrations for 36 h. The results indicated no colony formation after 10 d when the HeLa cells were treated with AM at the IC_50_ and IC_50_ concentrations for 36 h (Fig. 3c–d).

### Inhibition of Hela cell proliferation by AM

**Fig. 4:**
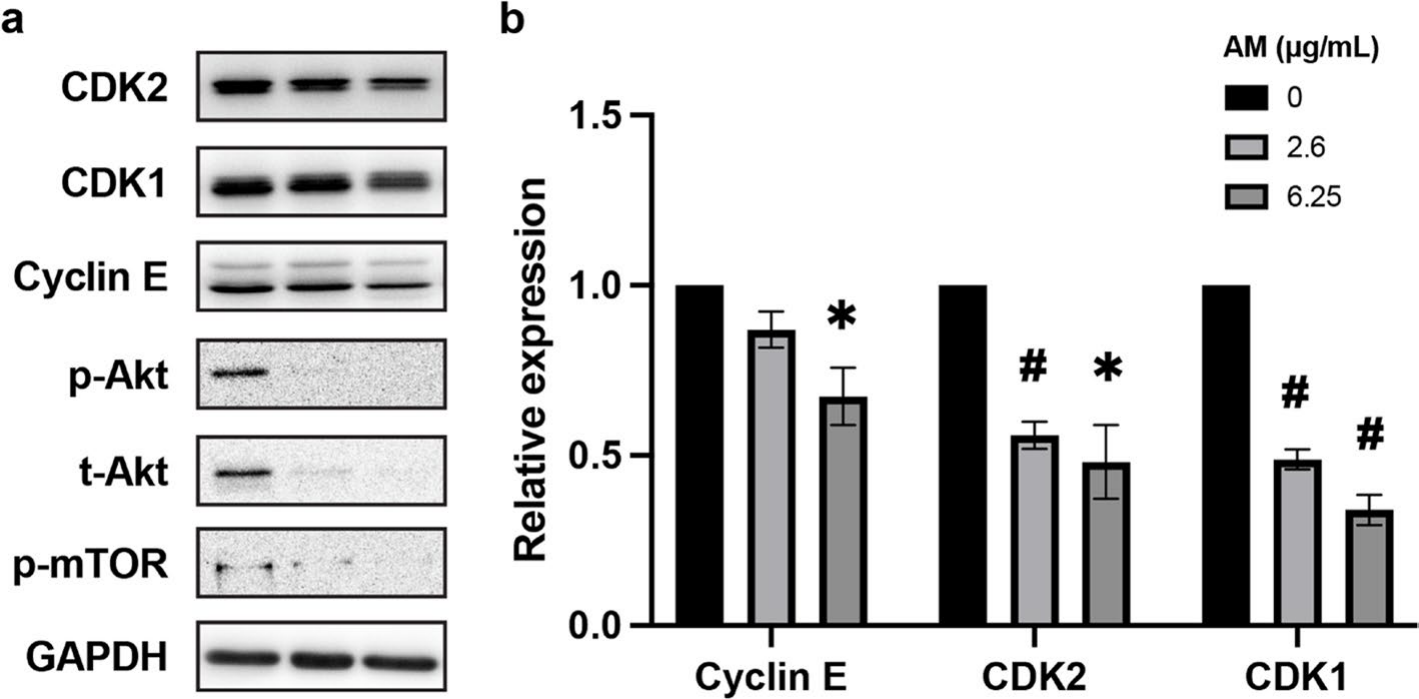
Effects of AM on cell proliferation in HeLa cells. **a** Western blot analysis displaying the reduced expression of cyclin E and CDK1/2 and the complete inhibition of expression of total-AKT, phospho-AKT, and phospho-mTOR upon treatment with IC_50_ (2.6 µg/mL) and IC_90_ (6.25 µg/mL) concentrations of AM for 12 h. The representative images are cropped from larger membranes. Original images of blots are shown in Fig. S7. **b** Relative band intensities of cyclin E and CDK1/2 in cells treated with AM to those of non-treated cells support the decrease in their expression upon AM treatment. Data are represented as mean ± SD and statistically analyzed by one-way ANOVA. Statistically significant differences were observed between the treated groups and the non-treated group; *, *p* < 0.05; #, *p* < 0.01; *n* = 3

The effects of AM on the cell cycle and its regulatory factors were examined by analyzing the expression levels of cyclin E, CDK1/2, AKT/mTOR pathway proteins, and intracellular ROS levels in HeLa cells treated with AM at IC_50_ or IC_90_ concentrations. Western blotting showed a decrease in cyclin E and CDK1/2 expression following treatment with AM. The expression of total AKT and phospho-AKT and its downstream target, phospho-mTOR, was considerably inhibited (Fig. 4).

### Activation of apoptosis by AM

**Fig. 5:**
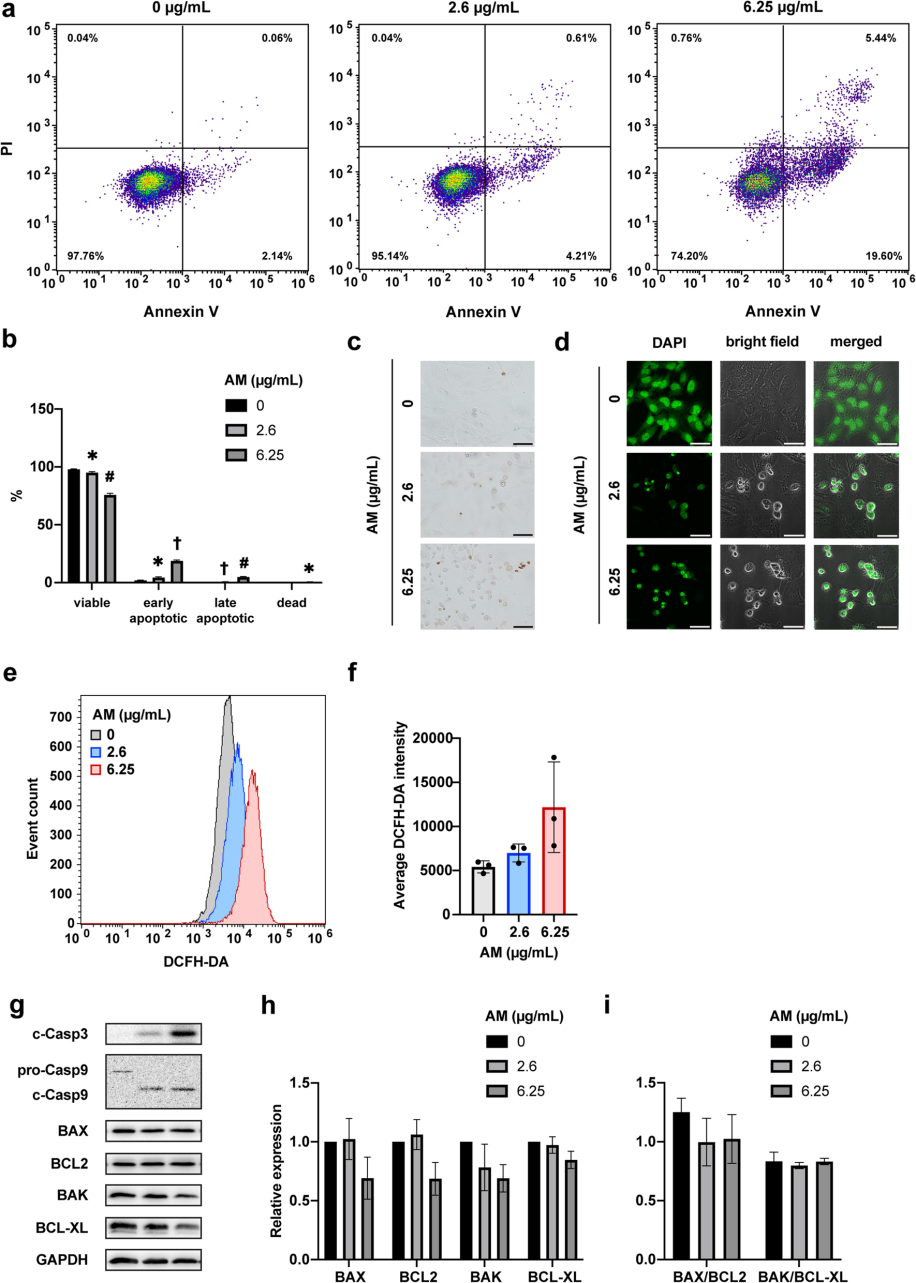
Analysis of pro-apoptotic effects of AM on HeLa cells. **a** Representative plots obtained by flow cytometry using Annexin A5 Apoptosis Detection kit were used to quantify cell viability and apoptosis following treatment with AM at IC_50_ and IC_90_ concentrations for 12 h. **b** Proportion of viable cells, early and late apoptotic cells, and dead cells analyzed using flow cytometry. **c** Representative TUNEL assay images captured at a magnification of 20 × , where brown cells indicate positive DNA fragmentation. **d** Fluorescent images of DAPI staining observed at 60 × magnification indicate nuclear fragmentation. **e** Flow cytometry using the DCFH-DA probe indicated the distribution of cell groups treated with AM at IC_50_ and IC_90_ concentrations compared with the non-treated cells, reflecting intracellular ROS levels in HeLa cells. **f** The average intensity of DCFH-DA supported the observed trend of increased ROS levels with AM treatment. **g** Western blot images of caspase 3, caspase 9, and pro-apoptotic (BAX, BAK) and anti-apoptotic markers (BCL2, BCL-XL) in HeLa cells treated with IC_50_ and IC_90_ doses of AM for 12 h. The representative images are cropped from larger membranes. Original images of blots are shown in Fig. S9. **h** Relative expression levels of pro-apoptotic (BAX, BAK) and anti-apoptotic markers (BCL2, BCL-XL) of AM-treated group to that of the non-treated group, calculated from the intensities of bands obtained by western blotting. **i** Ratio of expression levels of pro-apoptotic to those of anti-apoptotic proteins (BAX/BCL2 and BAK/BCL-XL). IC_50_, 2.6 µg/mL; IC_90_, 6.25 µg/mL. Data are represented as mean ± SD and statistically analyzed by one-way ANOVA. Statistically significant differences were observed between the treated and the non-treated groups; *, *p* < 0.05; #, *p* < 0.01; †, *p* < 0.001; *n* = 3. Scale bars indicate 50 μm

To investigate the effect of AM on apoptosis, we assessed the viability and apoptosis of HeLa cells by flow cytometry. Following 12 h of treatment with AM at IC_50_ or IC_90_ concentration, HeLa cells displayed decreased viability and increased numbers of early and late apoptotic cells (Fig. 5a, b). To detect DNA fragmentation, we performed the TUNEL assay, which revealed the presence of fragmented DNA in AM-treated HeLa cells (Fig. 5c). Additionally, DAPI staining was used to evaluate nuclear transformation, which showed nuclear shrinkage and fragmentation in the AM-treated cells (Fig. 5d). Flow cytometry using the DCFH-DA probe observed a tendency of an increase in intracellular ROS levels upon the AM concentration (Fig. 5e, f). Western blotting was performed to examine the activation of key apoptotic proteins, including caspase 3 and 9. The results showed the activation of both caspase 3 and 9 following treatment with AM (Fig. 5g). However, no noticeable signs of caspase 8 activation were observed (Fig. S8). The expression of both anti-apoptotic proteins (BCL2 and BCL-XL) and pro-apoptotic proteins (BAX and BAK) tended to decrease (Fig. 5h), whereas the ratios between BAX and BCL2, as well as BAK and BCL-XL (Fig. 5i), did not show marked changes.

## Discussion

Cervical cancer is a notable global health concern, particularly in LMICs where access to healthcare resources is limited. Therefore, newer therapeutic strategies, specifically natural and plant-derived compounds, are needed to treat this common cancer. Isolating numerous bioactive compounds from various parts of *S. affinis* highlights is potential for anticancer research. In this study, we investigated the anticancer properties of *S. affinis* fruit extract, focusing on the SAP and SAS components. Our findings revealed that the solvent fractions of the SAP extract did not exhibit notable cytotoxicity against HeLa cells. In contrast, the SAS fraction exhibited potent cytotoxic properties. The SAS-EA fraction displayed robust inhibition of HeLa cell proliferation, with an IC_50_ value of 2.62 ± 0.57 µg/mL. Notably, seeds of *Annona* species, which belong to the same family of *S. affinis*, Annonacae, have been previously reported as a rich source of anticancer agents [23]. This observation underscores the anticancer potential of specific plant tissues and warrants further investigation to develop effective plant-derived therapeutics.

Isolation of AM from the SAS-EA fraction further strengthens the significance of this study. Remarkably, the SA seeds contain approximately 3–12 times higher amounts of AM compared to other sources of *S. affinis,* such as root and flower [10, 18], not to mention other plants [24, 25]. Acetylmelodorinol demonstrated potent cytotoxic effects on HeLa cells in a dose-dependent manner, with an IC_50_ value of 2.62 ± 0.57 µg/mL. The IC_50_ value on HeLa cells was smaller, 1.55 times than that on OUMS-36 cells, highlighting the selective inhibition of AM on cancer cells compared to normal cells. The time-based assay results on HeLa cells showed suppressed cell viability from 4 h onwards at the IC_90_ concentration and 12 h onwards at the IC_50_ concentration. The clonogenic formation assay demonstrated strong inhibition of cell proliferation by AM after 36 h of exposure, with almost no colony formation after 10 d. Compared to a similar study, our findings demonstrated that AM exhibited stronger inhibition of colony formation in HeLa cells than in triple-negative breast cancer cells [21], suggesting its greater selectivity in affecting HeLa cells. Although this is not the first report of AM isolation [14, 15, 22, 24–26], further comprehensive studies are required to fully understand the precise molecular pathways involved in AM.

Cancer is a complex disease characterized by uncontrolled cell growth and proliferation, leading to unlimited cell survival ability [27]. Investigation of molecular pathways revealed that AM treatment inhibits the cell cycle and reduces cell survival via decreased expression of cyclin E and CDK1/2 on HeLa cells. Cyclin E and CDK1/2 are essential regulators of the cell cycle [28]. The reduction in the levels of these factors observed with AM treatment suggests its potential to act as a cell cycle inhibitor to inhibit cancer cell growth and progression. The AKT/mTOR pathway is crucial for promoting cell survival [29], and its activation is associated with resistance to various cancer therapies and poor prognosis [30]. This study observed AM’s inhibition of the AKT/mTOR pathway at the IC_50_ and IC_90_ concentrations, indicating its potential to activate cell death mechanisms in HeLa cells.

Our study utilized Annexin V apoptosis analysis to reveal a notable increase in early and late apoptotic cells following treatment with AM. The apoptosis process is characterized by specific markers [31–33]. First, ROS levels play crucial roles in physiological functions such as cell cycle progression and proliferation [32]. However, the elevated levels of intracellular ROS detected in this study upon exposure of HeLa cells to AM can trigger apoptosis. We also observed DNA fragmentation and nuclear disintegration, recognized as established markers for apoptosis [31, 33]. At the molecular level, the activation of caspases 3 and 9 indicated AM’s tendency to induce pro-apoptotic behavior. Caspase 8 and caspase 9, acting as initiator caspases in the extrinsic and intrinsic apoptosis pathways, respectively, showcased distinctive activation patterns [34]. Among all caspases, caspase 3 is a crucial executioner in both pathways [35]. Our study revealed the activation of caspase 9 but not caspase 8, indicating that AM triggers the intrinsic apoptotic pathway. This finding contrasts with a previous study conducted on different tumor cells (MDA-MB-231 cells), which demonstrated that AM activates apoptosis in both pathways by elevating cleaved caspase 8 [21]. These findings imply the activation of the loss of OMM potential. This can be attributed to the upregulation and translocation of BAX from the cytosol to the OMM, along with the inhibition of BCL-2, resulting in an increased BAX/BCL-2 ratio [7]. Other reports mentioned that the loss of permeability of OMM was induced by the decreased expression level of anti-apoptotic proteins BCL-2 and BCL-XL [36, 37]. Therefore, AM may induce OMM permeabilization in HeLa cells via a reduction in the expression levels of the anti-apoptotic proteins BCL-2 and BCL-XL, although the BAX/BCL-2 and BAK/BCL-XL ratios did not show an increase in this study. This observation aligns with a previous report discussing the impact of reduced BCL-2 expression on OMM loss [38].

## Conclusions

In summary, this study contributes to our understanding of the anticancer potential of *S. affinis* fruit, particularly the promising cytotoxic effects of AM isolated from the seed fraction. The SAS appears to be the primary source of anticancer properties of *S. affinis* fruit. AM has been found to hinder cell proliferation, and compelling evidence indicates its ability to induce apoptosis in cancer cells. However, the exact mechanism by which AM activates apoptosis remains unclear. Therefore, additional investigation is required to understand the molecular pathways involved comprehensively. While these findings represent a significant advancement in our knowledge of the anticancer capabilities of *S. affinis* fruit, further research and validation are imperative to fully harness their therapeutic potential.

### Supplementary Information

**Additional file 1: Fig. S1.** Acetylmelodorinol in CDCl3 1H NMR at 298 K. **Fig. S2.** Acetylmelodorinol in CDCl3 13C NMR at 298 K. **Fig. S3.** Acetylmelodorinol in CDCl3 DQF-COSY at 298 K. **Fig. S4.** Acetylmelodorinol in CDCl3 NOESY at 298 K. **Fig. S5.** Acetylmelodorinol in CDCl3 1H-13C HSQC at 298 K. **Fig. S6.** Acetylmelodorinol in CDCl3 1H-13C HMBC at 298 K. **Fig. S7.** Original images of blots shown in Fig. 4a. **Fig. S8.** Western blottingof caspase-8. **Fig. S9.** Original images of blots shown in Fig. 5a.

**Additional file 2: Table S1. **Antibodies for Western Blot Assay.

## Data Availability

The datasets generated and/or analyzed during the current study are available from the corresponding author on reasonable request.

## Abbreviations

ANOVA: Analysis of variance
AKT: Protein kinase B
BAK: BCL2 antagonist/killer
BAX: BCL2 associated X
BCL2: B-cell lymphoma 2
BCL-XL: B-cell lymphoma-extra large
CCK8: Cell counting kit-8
CDKs: Cyclin-dependent kinases
DAPI: 4′,6-Diamidino-2-phenylindole
HPV: Human papillomavirus
IC_50_: Half-maximal inhibitory concentrations
IC_90_: 90% of the maximal inhibitory concentration
mTOR: Mammalian target of rapamycin
OMM: Mitochondrial outer membrane
ROS: Reactive oxygen species
SAP: *S. affinis* pericarp
SAS: *S. affinis* seed
SD: Standard deviation
TUNEL: Terminal deoxynucleotidyl transferase dUTP nick end labeling
