# Supplementary material for: Acetylmelodorinol isolated from Sphaerocoryne affinis seeds inhibits cell proliferation and activates apoptosis on HeLa cells
Source: BMC Complement Med Ther. 2024 Jan 27;24:59. doi: 10.1186/s12906-024-04357-w (PMC10821558; doi:10.1186/s12906-024-04357-w)
Supplement: Supplementary file 1 — Additional file 1: Fig. S1. Acetylmelodorinol in CDCl3 1H NMR at 298 K. Fig. S2. Acetylmelodorinol in CDCl3 13C NMR at 298 K. Fig. S3. Acetylmelodorinol in CDCl3 DQF-COSY at 298 K. Fig. S4. Acetylmelodorinol in CDCl3 NOESY at 298 K. Fig. S5. Acetylmelodorinol in CDCl3 1H-13C HSQC at 298 K. Fig. S6. Acetylmelodorinol in CDCl3 1H-13C HMBC at 298 K. Fig. S7. Original images of blots shown in Fig. 4a. Fig. S8. Western blottingof caspase-8. Fig. S9. Original images of blots shown in Fig. 5a. [file 12906_2024_4357_MOESM1_ESM.pdf]

Fig. S1: Acetylmelodorinol in $CDCl_3$ ^1H NMR at 298 K

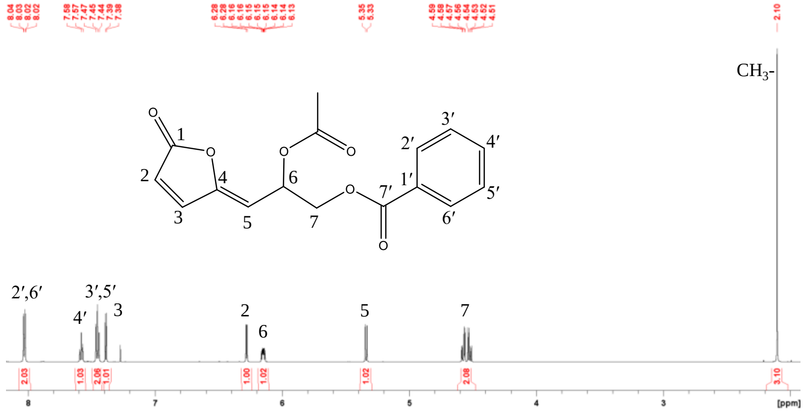

Fig. S2: Acetylmelodorinol in $CDCl_3$ ^{13}C NMR at 298 K

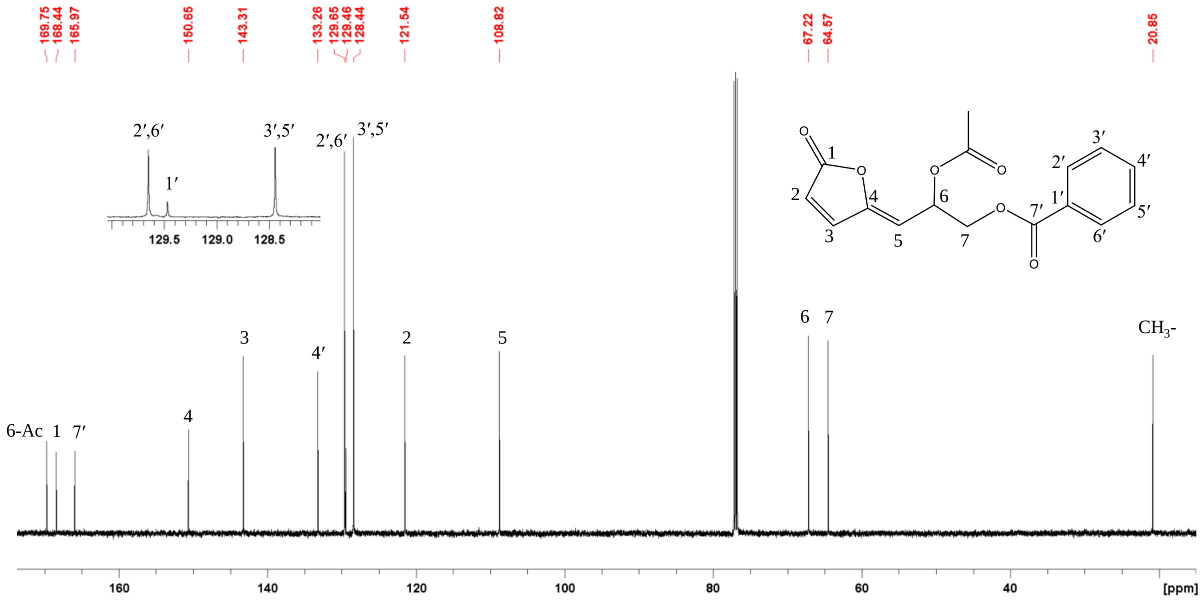

Fig. S3: Acetylmelodorinol in $CDCl_3$ DQF-COSY at 298 K

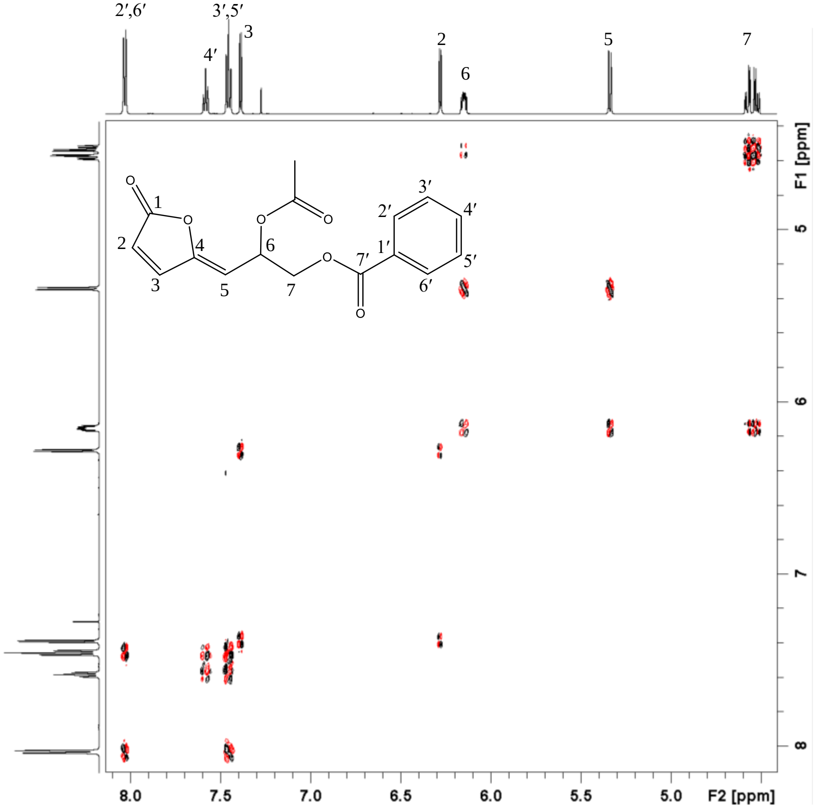

Fig. S4: Acetylmelodorinol in $CDCl_3$ NOESY at 298 K

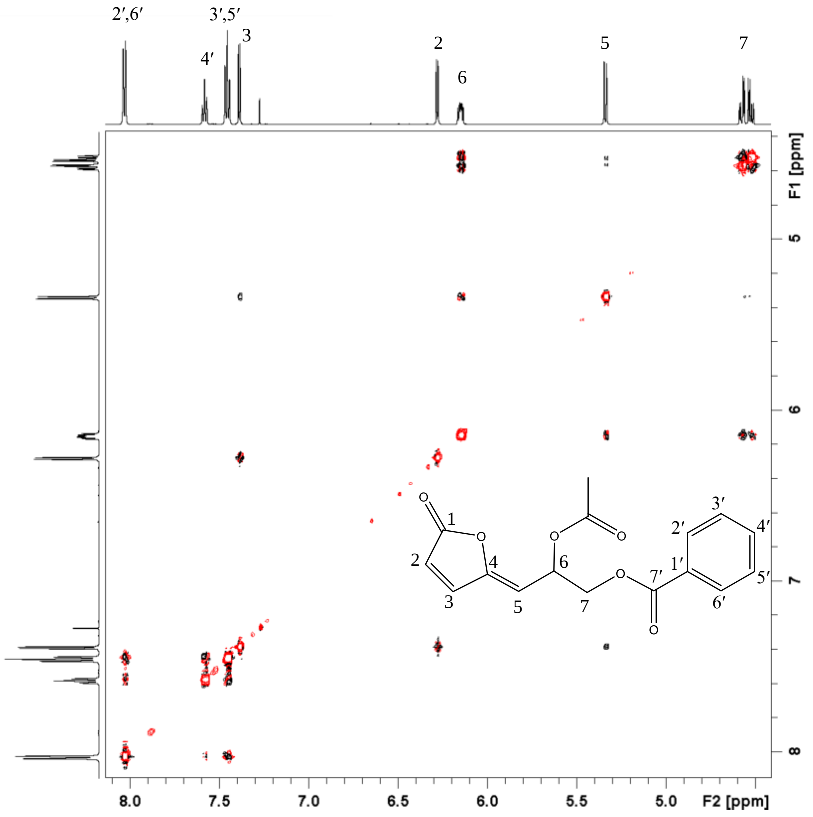

Fig. S5: Acetylmelodorinol in $CDCl_3$ ^1H-^{13}C HSQC at 298 K

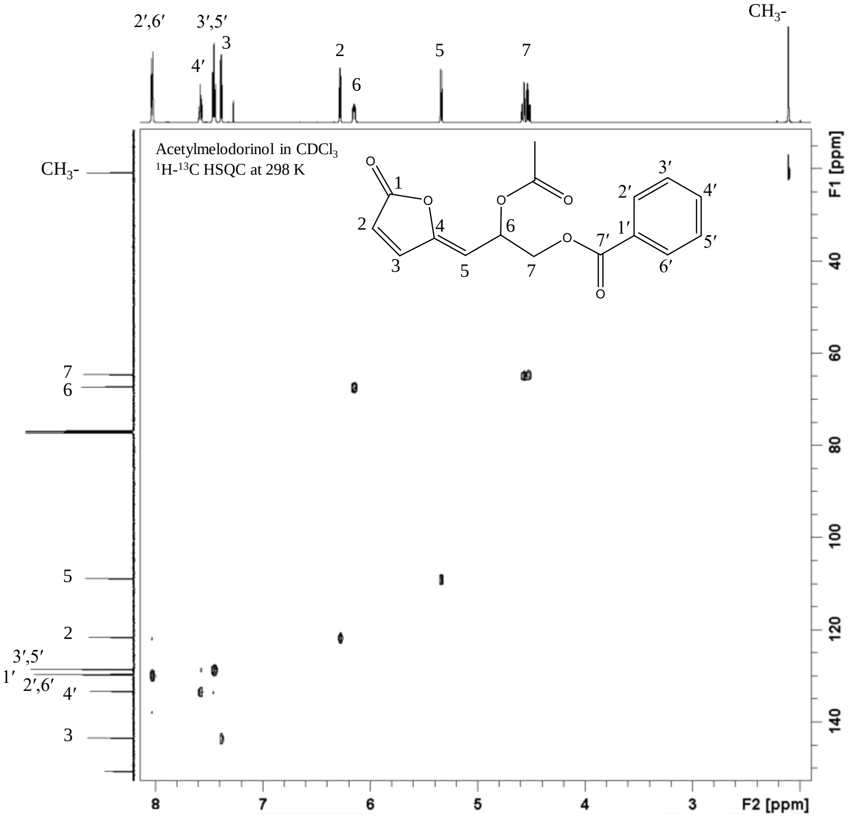

Fig. S6: Acetylmelodorinol in $CDCl_3$ ^1H-^{13}C HMBC at 298 K

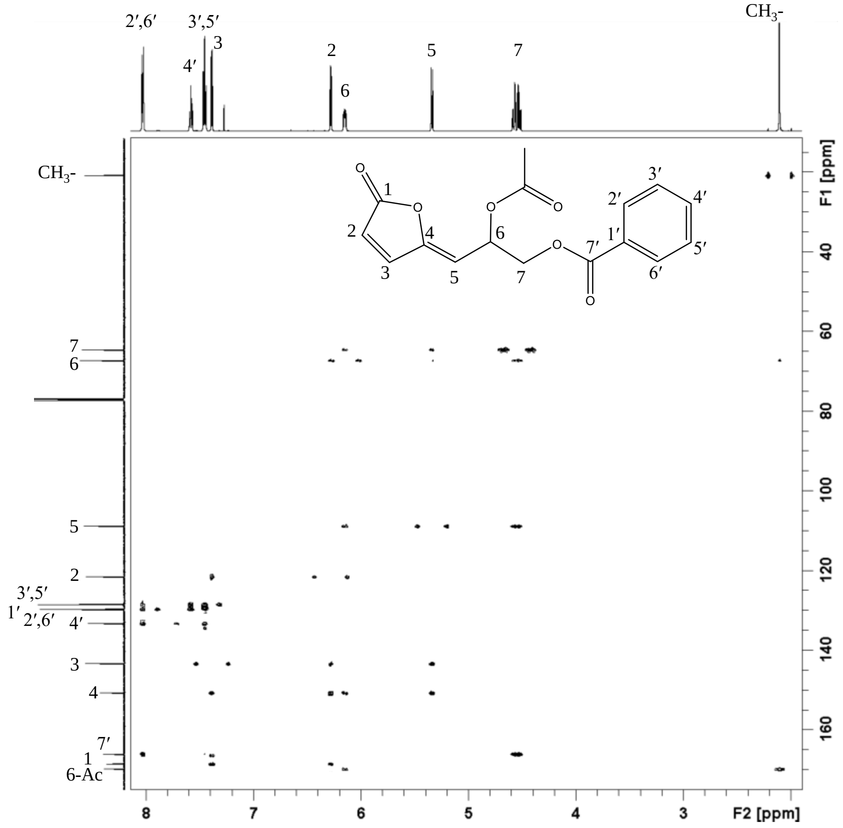

Fig. S7 Original images of blots shown in Fig. 4a

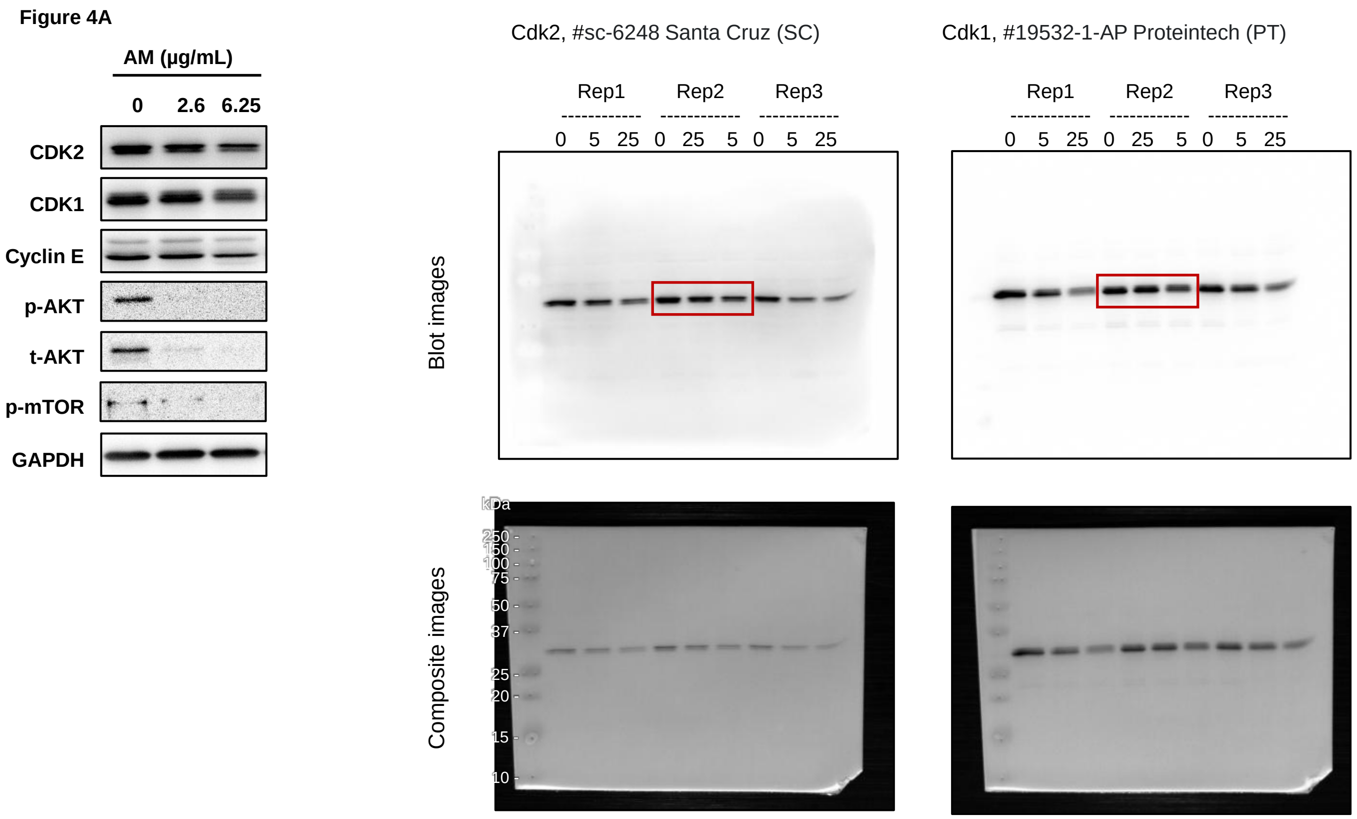

Boxed bands are shown in Fig. 4a as representives.

Fig. S7 (continued) Original images of blots shown in Fig. 4a

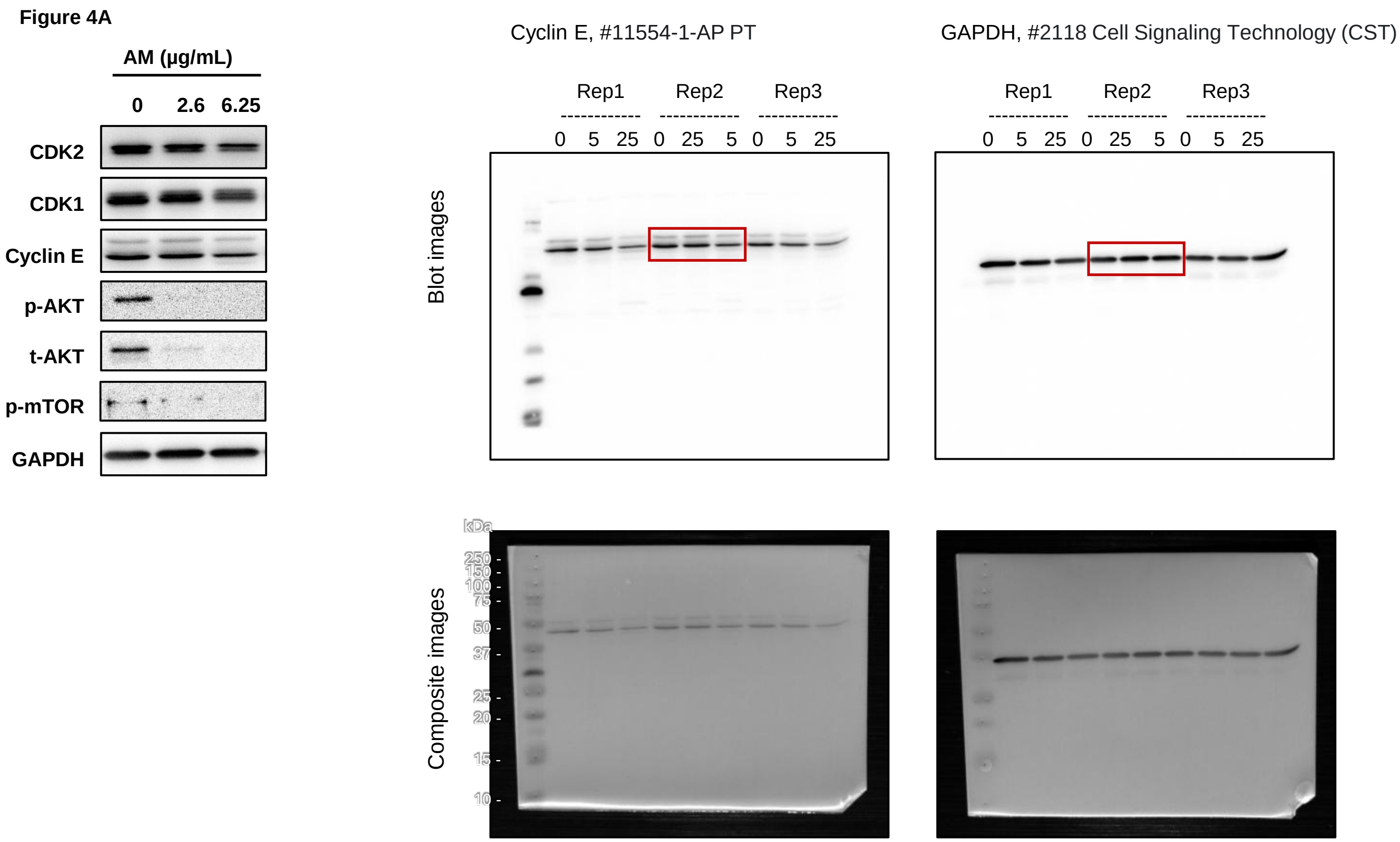

Boxed bands are shown in Fig. 4a as representives.

Fig. S7 (continued) Original images of blots shown in Fig. 4a

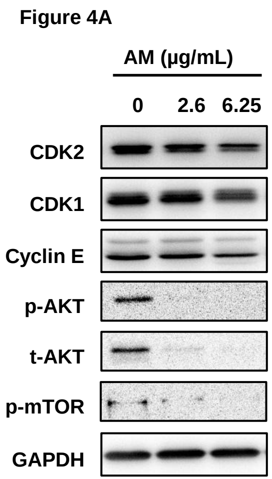

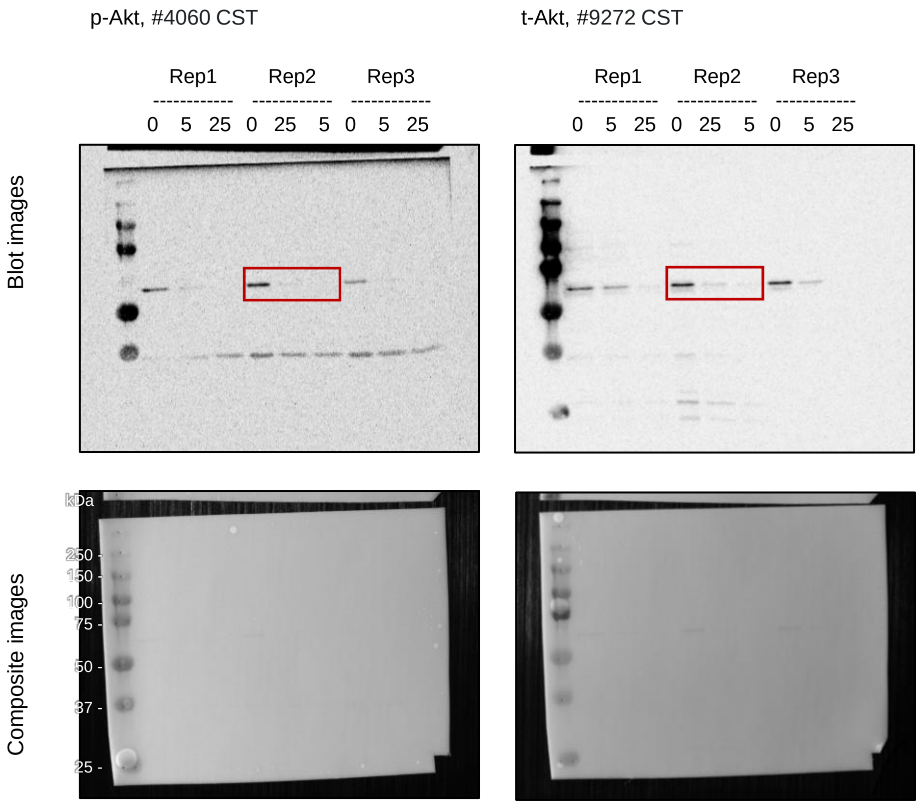

Boxed bands are shown in Fig. 4a as representives.

Fig. S7 (continued) Original images of blots shown in Fig. 4a

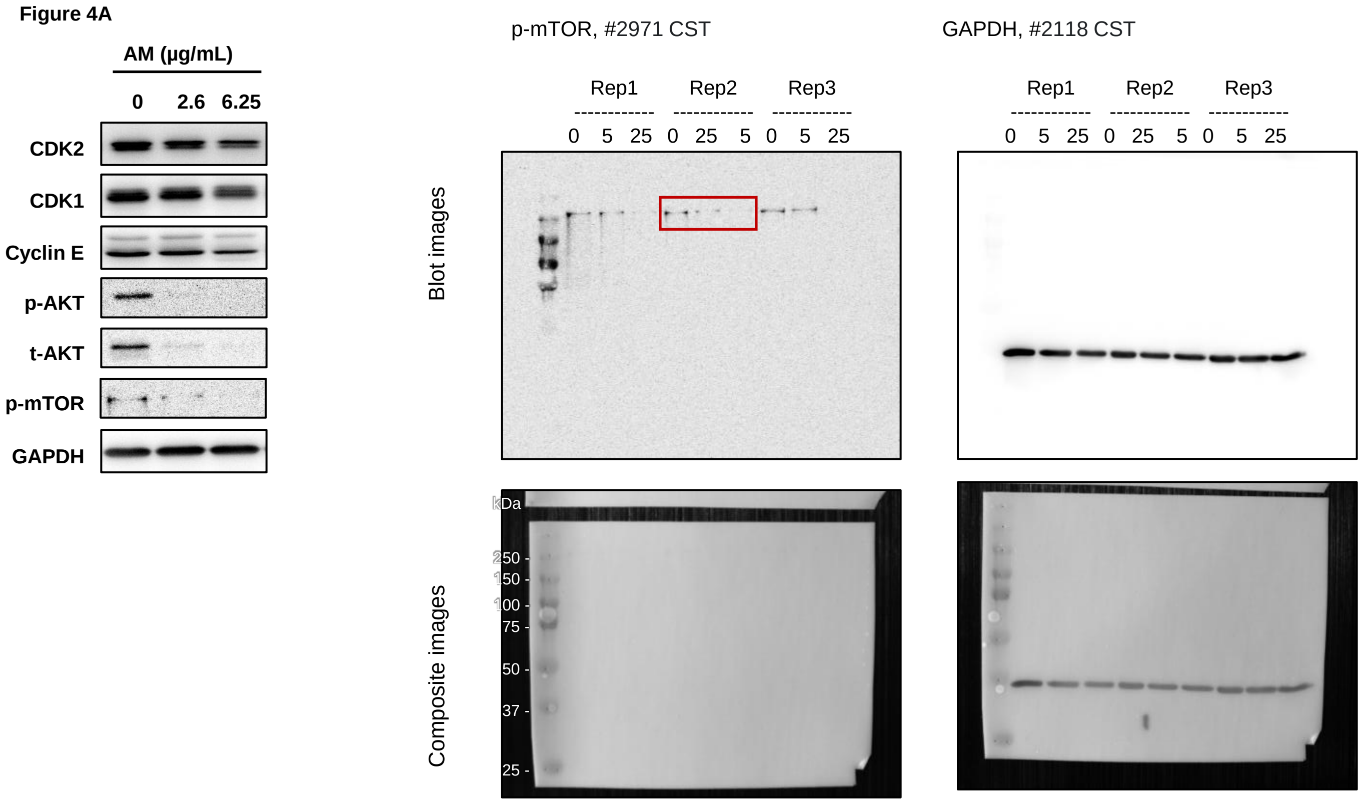

Boxed bands are shown in Fig. 4a as representives.

Fig. S8: Western blotting of caspase-8

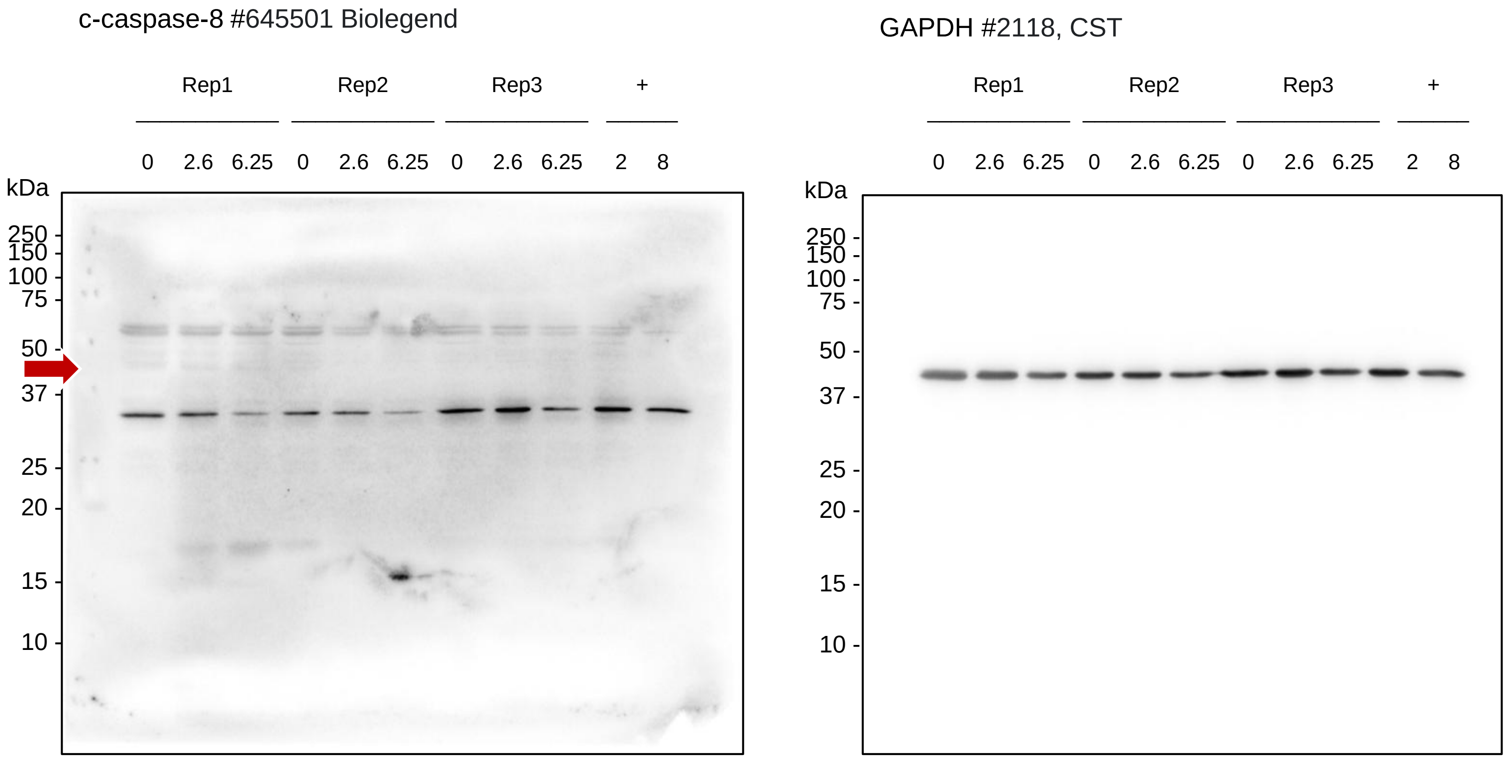

+, Staurosporine 1 µM; 2, 2 h; 8, 8 h
The red arrow indicates caspase-8.

Fig. S9 Original images of blots shown in Fig. 5a

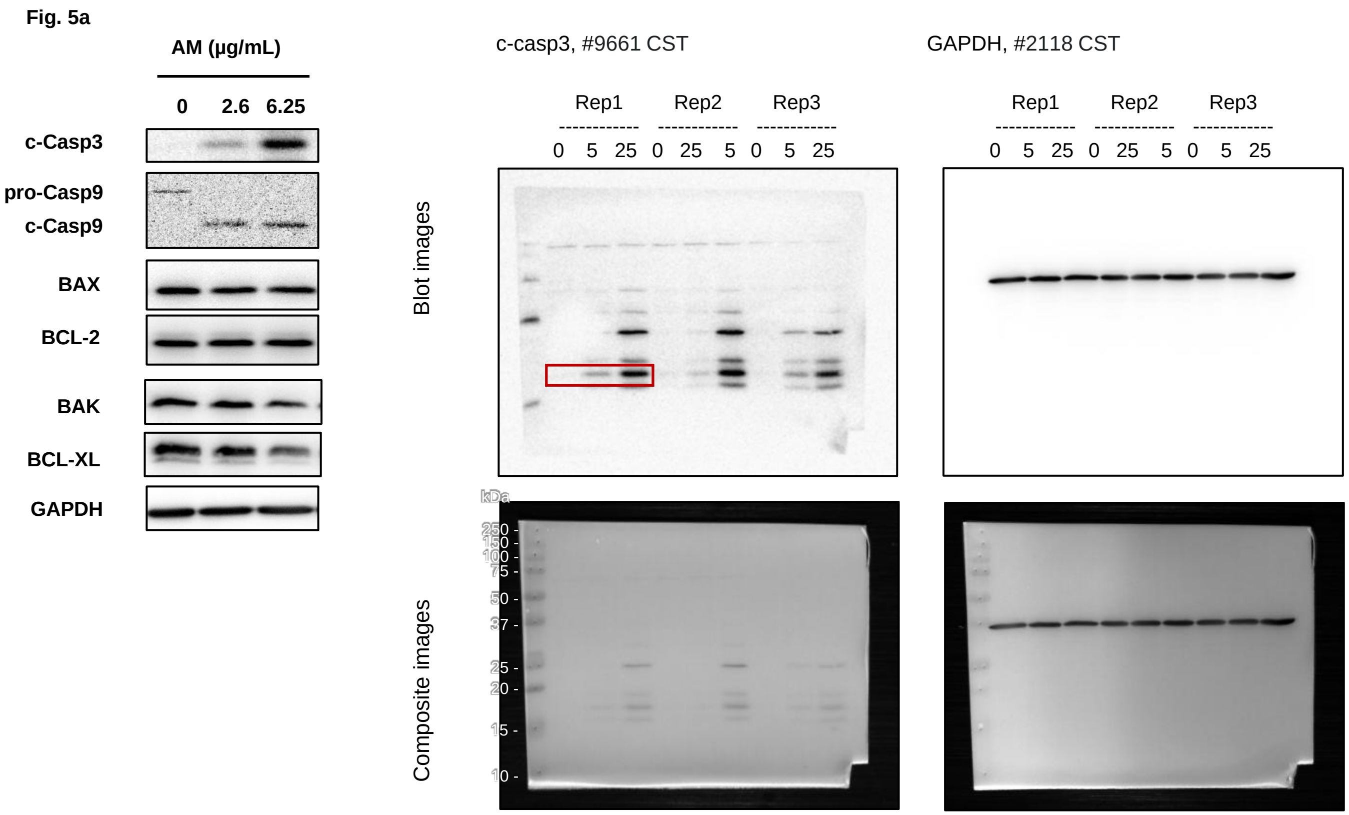

Boxed bands are shown in Fig. 5a as representives.

Fig. S9 (continued) Original images of blots shown in Fig. 5A

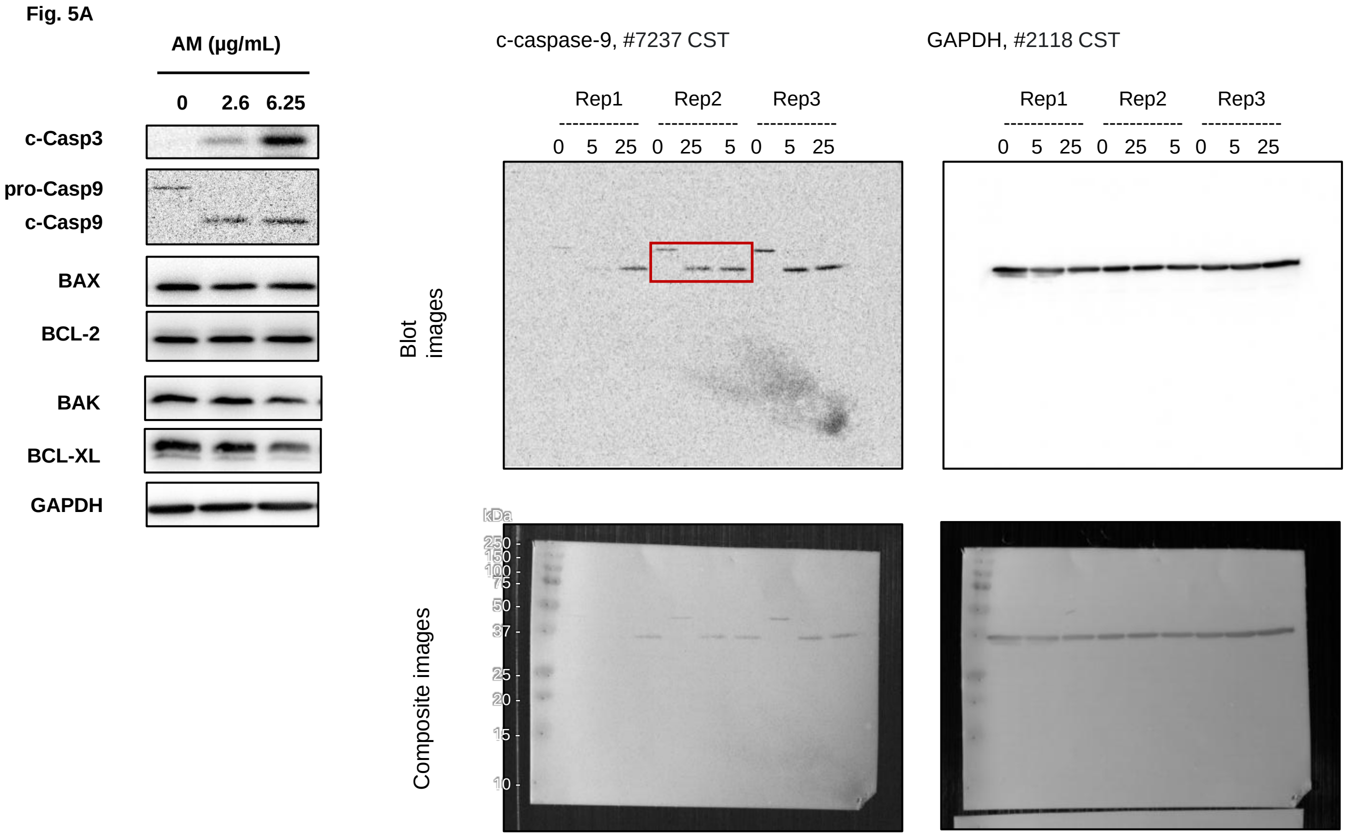

Boxed bands are shown in Fig. 5a as representives.

Fig. S9 (continued) Original images of blots shown in Fig. 5A

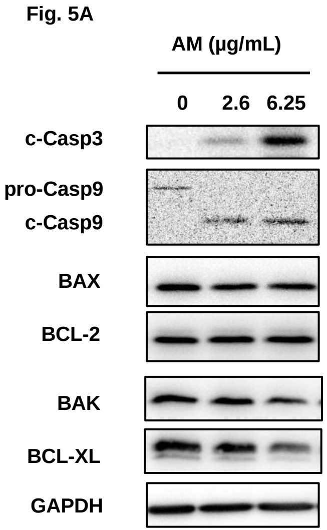

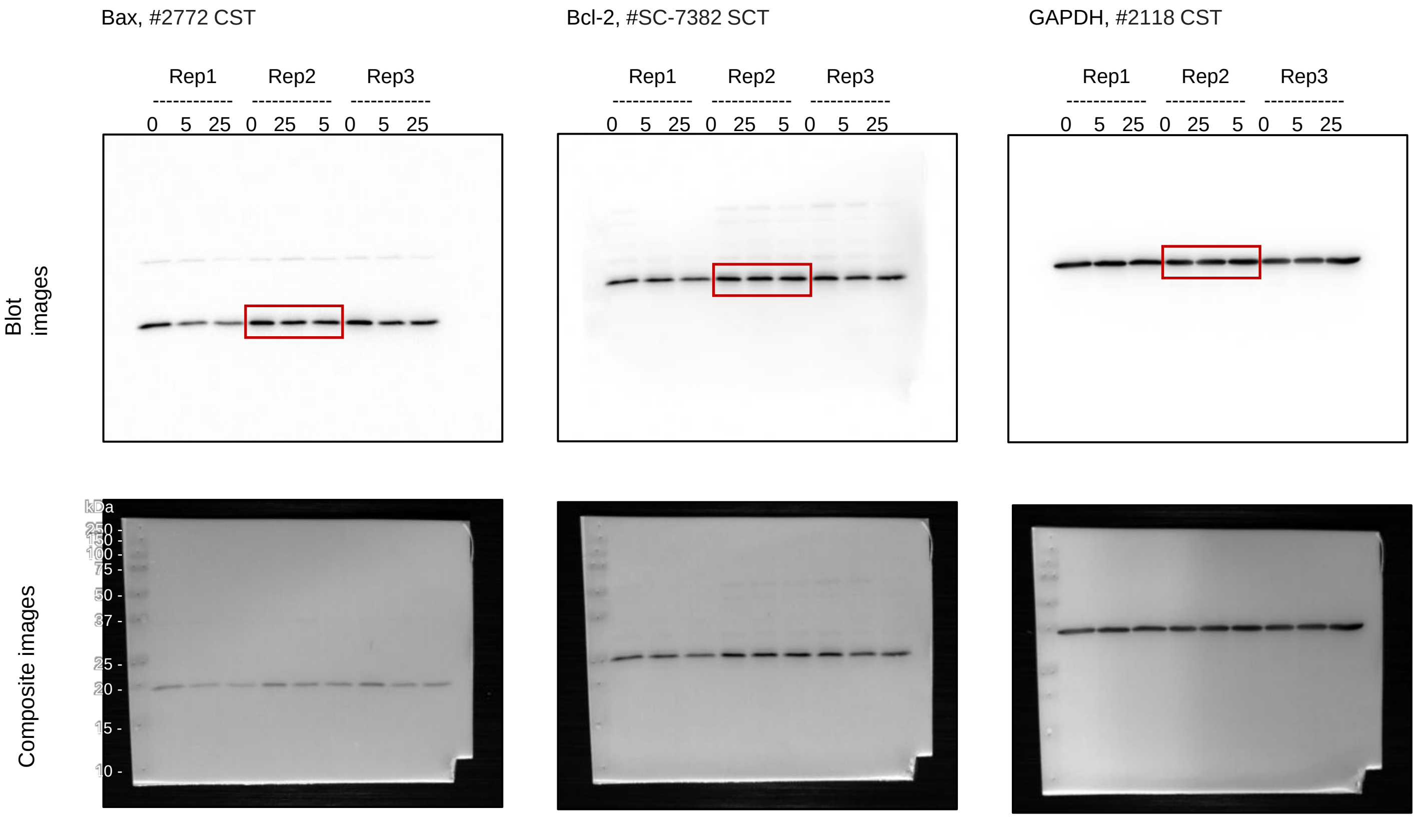

Boxed bands are shown in Fig. 5a as representives.

Fig. S9 (continued) Original images of blots shown in Fig. 5A

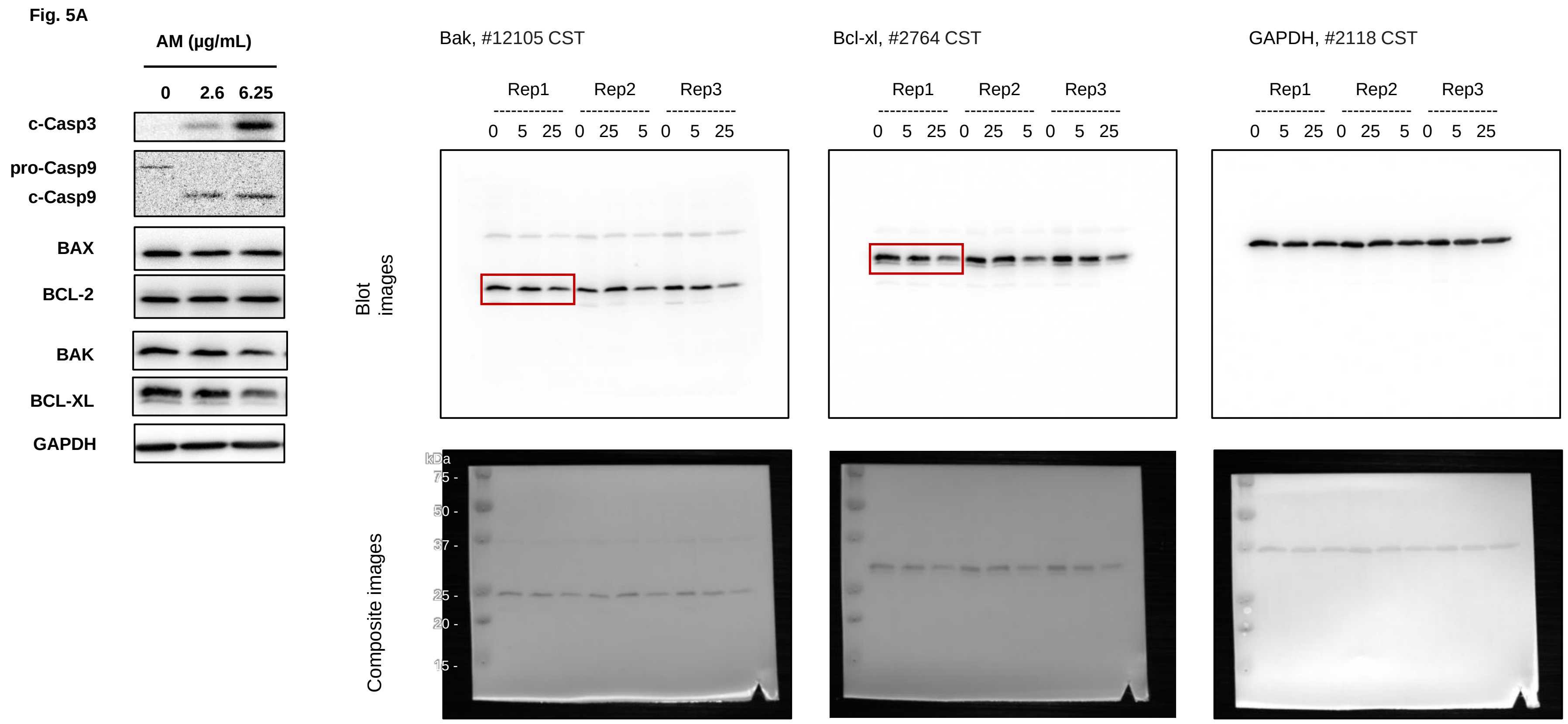

Boxed bands are shown in Fig. 5a as representives.
