# Supplementary material for: Acetylmelodorinol isolated from Sphaerocoryne affinis seeds inhibits cell proliferation and activates apoptosis on HeLa cells
Source: BMC Complement Med Ther. 2024 Jan 27;24:59. doi: 10.1186/s12906-024-04357-w (PMC10821558; doi:10.1186/s12906-024-04357-w)
Supplement: Supplementary file 2 — Additional file 2: Table S1. Antibodies for Western Blot Assay. [file 12906_2024_4357_MOESM2_ESM.pdf]

Table S1. Antibodies for Western Blot Assay

Antibody	Dilution rate	Code	Manufacturer
Anti-BAX	1:1000	2772	Cell Signaling
Anti-BCL-2	1:1000	SC-7382	Santa Cruz Biotech
Anti-c-Casp3	1:1000	9661	Cell Signaling
Anti-c-Casp9	1:1000	7237	Cell Signaling
Anti-Cyclin E	1:4000	11554-1-AP	Proteintech
Anti-CDK2	1:1000	SC-6248	Santa Cruz Biotech
Anti-CDK1	1:1000	19532-1-AP	Proteintech
Anti-p-AKT	1:1000	4060	Cell Signaling
Anti-t-AKT	1:1000	9272	Cell Signaling
Anti-p-mTOR	1:1000	2971	Cell Signaling
Anti-GAPDH	1:4000	2118	Cell Signaling
